# A therapeutic barium enema is a practical option to control bleeding from the appendix

**DOI:** 10.1186/1471-230X-13-152

**Published:** 2013-10-25

**Authors:** Youkou Konno, Mikihiro Fujiya, Kazuyuki Tanaka, Aki Sakatani, Mizue Shimoda, Akihiro Hayashi, Momotaro Muto, Mitutaka Inoue, Jun Sakamoto, Kensuke Oikawa, Nobuhiro Ueno, Yuhei Inaba, Kentaro Moriichi, Yutaka Kohgo

**Affiliations:** 1 Department of Internal Medicine, Engaru-Kosei general Hospital, Engaru, Hokkaido 099-0404, Japan; 2 Division of Gastroenterology and Hematology/Oncology, Department of Medicine, Asahikawa Medical University, 2-1 Midorigaoka-higashi, Asahikawa, Hokkaido 078-8510, Japan; 3 Department of Gastroenterology, Sapporo Higashi Tokushukai Hospital, Sapporo, Hokkaido 065-0033, Japan; 4 Department of pathology, Asahikawa-Kosei general Hospital, Asahikawa, Hokkaidō 078-8211, Japan

**Keywords:** Appendix bleeding, Barium enema, Intestinal hemorrhage, Appendicitis

## Abstract

**Background:**

Acute lower gastrointestinal hemorrhage originating from the appendix is rare and often intractable, because it is almost impossible to approach the bleeding point by endoscopy. We herein describe the first case of bleeding from the appendix, which was successively controlled by a therapeutic barium enema administered into the appendix.

**Case presentation:**

A 71-year-old male visited our hospital because of melena. He has been receiving an anti-coagulation drug, ticlopidine hydrochloride, for 10 years. By an emergency colonoscopy, a hemorrhage was detected in the appendix, and the lesion responsible for the bleeding was regarded to exist in the appendix. Two hundred milliliters of 50 W/V% barium was sprayed into the orifice of the appendix using a spraying tube. The bleeding could thus be immediately stopped, and a radiological examination revealed the accumulation of barium at the cecum and the orifice of the appendix. The barium accumulation disappeared by the next day, and no obvious anal bleeding was observed. Two weeks after stopping the bleeding from the appendix, an appendectomy was performed to prevent any further refractory hemorrhaging. The patient has had no complaints of any abdominal symptoms or anal bleeding for 10 months.

**Conclusions:**

A therapeutic barium enema is a useful procedure to control bleeding from the appendix and to avoid emergency surgery, such as partial cecectomy and hemicolectomy.

## Background

Acute lower gastrointestinal hemorrhage originating from the appendix is rare and often intractable [1-22], because it is very difficult to approach the bleeding point in the appendix by endoscopy. Therefore, in most cases with bleeding from the appendix, an emergency operation was necessary [1-13,15-22]. We herein report the first case of the bleeding associated with the appendix ulcer which was successively controlled by the administration of a therapeutic barium enema into the appendix.

## Case presentation

**Figure 1:**
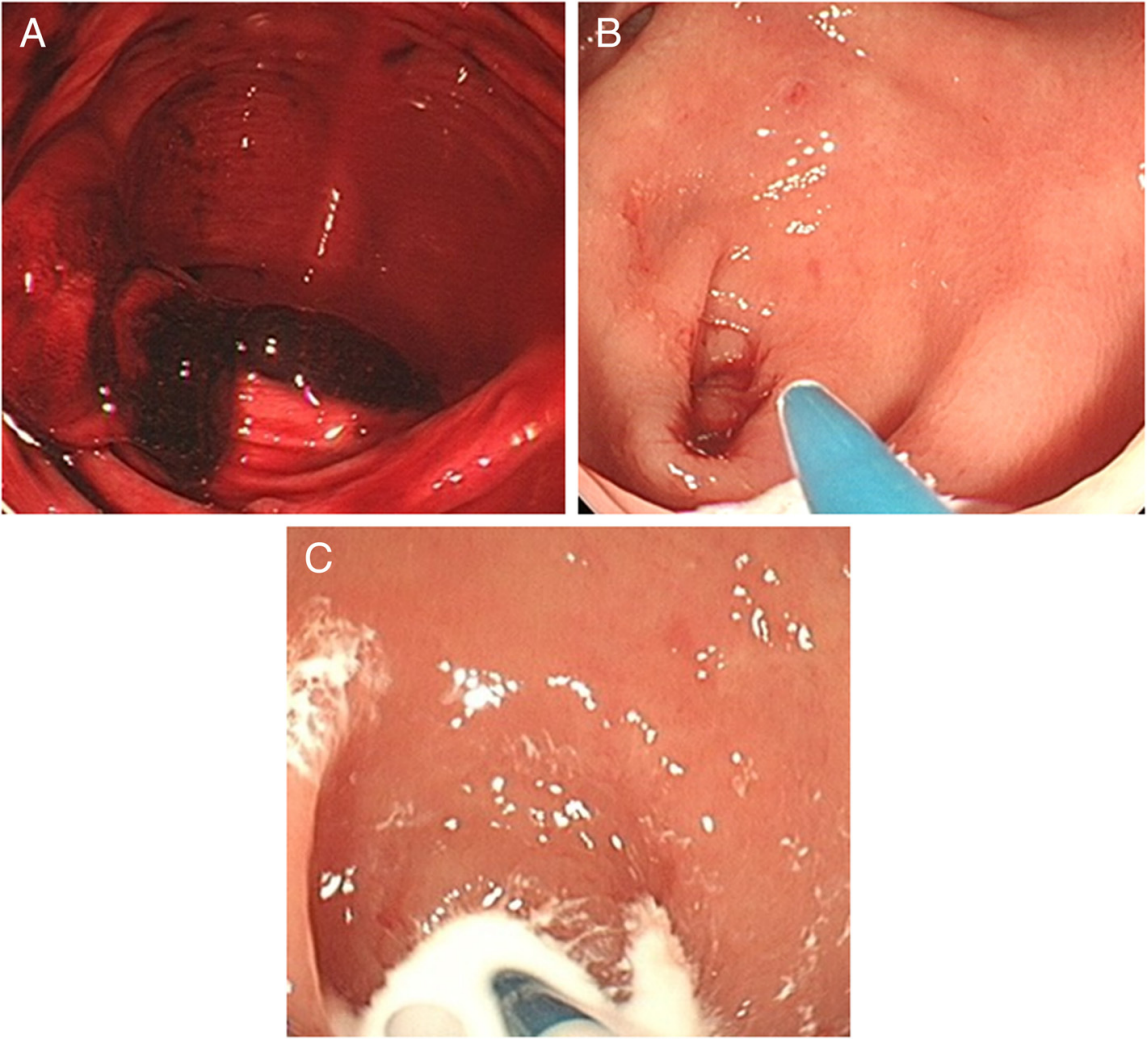
**The findings of emergency endoscopy.** Blood and coagula were observed in the cecum **(A)**. After washing with water, a hemorrhage from the appendix was detected **(B)**. Two hundred ml of 50 W/V% barium was sprayed into the orifice of the appendix through a spraying tube **(C)**.

**Figure 2:**
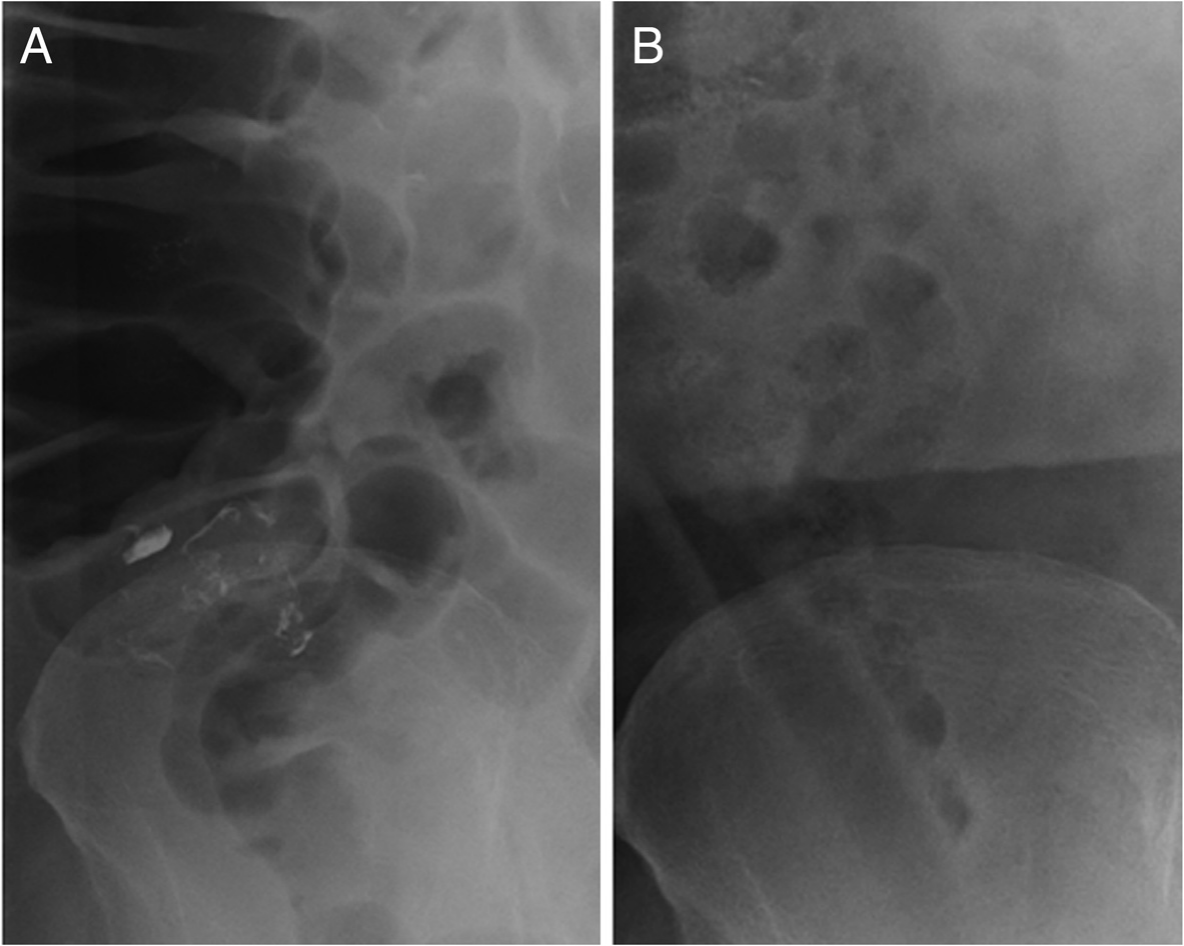
**Radiological examinations of the abdomen.** A radiological examination showed the accumulation of the barium at the cecum and orifice of the appendix **(A)**. By the next day, the accumulation had almost completely disappeared **(B)**.

**Figure 3:**
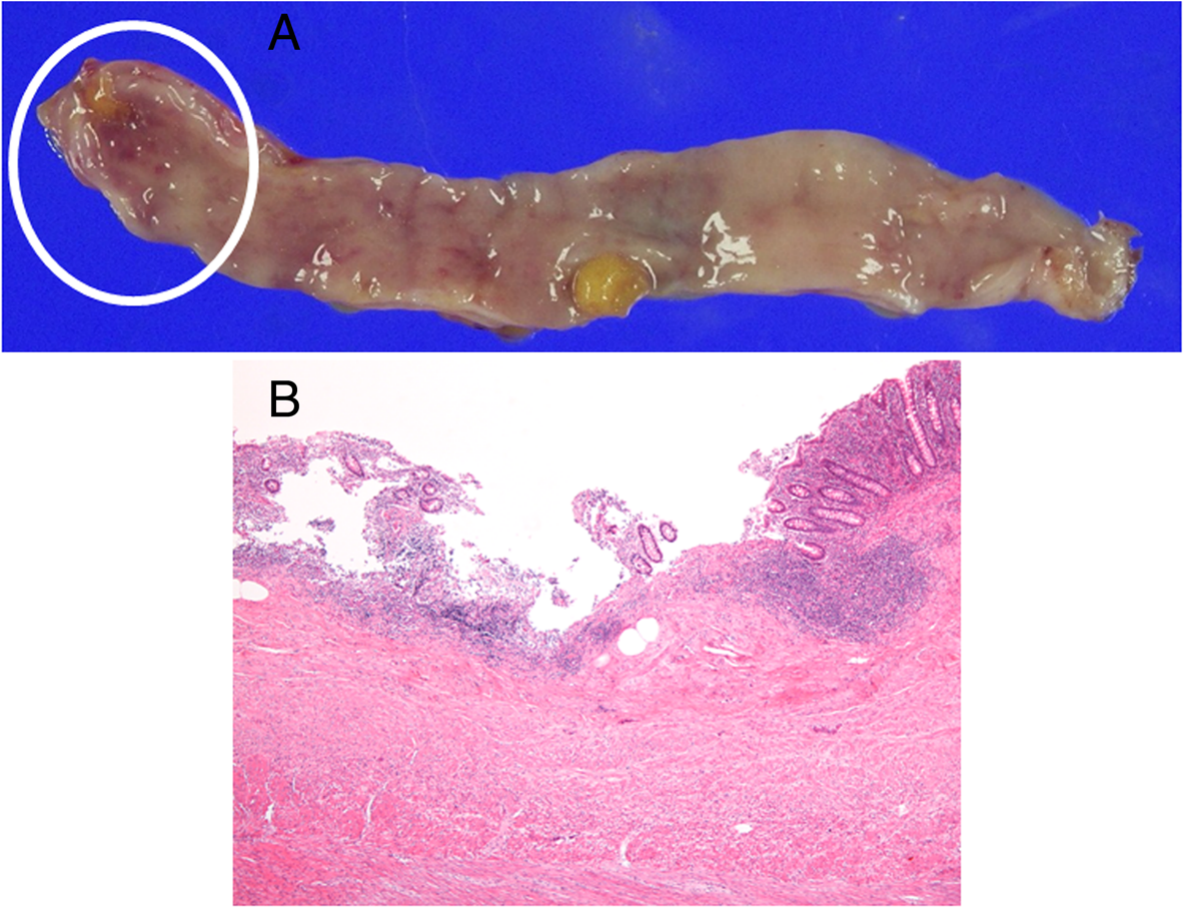
**Histological features of the surgical specimen.** A gross surgical specimen showed an ulcer in the tip of the appendix with no tumorous lesion **(A)**. The histological sections revealed a severe infiltration of neutrophils and lymphocytes within a shallow ulcer (Hematoxilin eosin staining, ×40) **(B)**.

A 71-year-old male visited our hospital because of melena. He was suffering from diabetes mellitus and the sequelae of a cerebral infarction. He has been receiving oral diabetic drugs and an anti-coagulation drug, ticlopidine hydrochloride for 10 years. His blood pressure was 150/83 mmHg and the pulse rate was 105/minute. A blood examination revealed a high level of blood sugar at 162 mg/dL, and hemoglobin A1c of 6.6%, but neither a decrease in red blood cells nor hemoglobin. A computed tomography scan in the abdomen detected a high density fluid of ascites, but no inflammatory changes in any organs, including the intestinal tract. By an emergency colonoscopy, the fresh blood in the entire colon, but not in the ileum, and the blood and coagula in the orifice of the appendix were observed (Figure 1A). After washing the orifice with water, a hemorrhage was detected in the appendix (Figure 1B), and the lesion responsible for bleeding was regarded to exist in the appendix. After obtaining the patient’s informed consent, 200 ml of 50 W/V% barium was sprayed into the orifice of the appendix using a spraying tube (Figure 1C), because a therapeutic barium enema has been shown to be a useful procedure to control bleeding from a diverticulum [23,24]. The bleeding could be immediately stopped, and a radiological examination revealed the accumulation of barium at the cecum and the orifice of the appendix (Figure 2A). The barium accumulation disappeared by the next day (Figure 2B) and no obvious anal bleeding was observed in the patient. Two weeks after stopping the bleeding from the appendix, an appendectomy was performed to prevent further refractory hemorrhage from the appendix. A gross specimen showed ulceration, but no tumorous lesions, at the tip of the appendix (Figure 3A). A severe infiltration of neutrophils and lymphocytes within a shallow ulcer was histologically observed (Figure 3B). The administration of the anti-coagulant ticlopidine hydrochloride, was restarted two weeks after the operation. The patient has had no complaints of any abdominal symptoms, including anal bleeding, for 10 months.

## Discussion and conclusion

This report presented the first case of bleeding from an appendix ulcer that was successfully controlled with therapeutic barium enema. To date, 23 cases of bleeding from the appendix have been reported [1-22] (Table 1). The age of the patients with bleeding from the appendix ranged from 9 to 76 years of age. Sixteen patients were male, while the others were female. The causes of the bleeding included diverticulitis [1], Crohn’s disease [2,4,16], a mucinous cyst [6], aorta-appendix fistula [15], ectopic uterus mucosa [10], angiodysplasia [11,14], intussusceptions [3,8,9,13], gastrointestinal stromal tumor [20] and intestinal tuberculosis [21]. Anti-coagulation drugs were administered in 4 cases [5,17,20]. In the present case, the administration of the anti-coagulation drug, ticlopidine hydrochloride, was thought to aggravate the bleeding from the appendix, while the cause of the ulceration itself of the appendix remains unclear. The therapeutic barium enema is thought to have stopped the acute bleeding from the appendix, and the suspension of the administration of the anti-coagulant helped to prevent re-bleeding. In many of the reported cases, the appendix bleeding has been diagnosed via colonoscopy.

**Table 1:** The reported cases and our case of bleeding from appendix

**Authors**	**Age**	**Sex**	**Administration of anti-coagulation drugs**	**Method of diagnosis**	**Treatment**	**Histopathologic findings**
Tamvakopoulos (1969) [1]	40	M		Not described	Conventional appendectomy	Diverticulitis
Tamvakopoulos (1969) [1]	43	F		Barium enema	Conventional appendectomy	Diverticulitis
Geerken and Gibbons (1974) [2]	17	M		Barium enema	Conventional appendectomy	Crohn’s disease
Brewer and Wangensteen (1974) [3]	24	F		Barium enema	Ileocecal resection	Intussusception
Brown and Peter (1976) [4]	19	M		Barium enema	Right hemicolectomy	Crohn’s disease
Milewski (1977) [5]	14	M	Aspirin tablet taken on the night of admission	Not described	Ileocecal resection	Appendicitis, abscess
Mullen (1979) [6]	63	M		Barium enema	Right hemicolectomy	Diverticulum, mucocele
Norman et al (1980) [7]	48	M		Angiography	Conventional appendectomy	Diverticulum
McIntosh et al (1990) [8]	18	F		CF, CT	Conventional appendectomy	Intussusception
Jevon et al (1992) [9]	32	F		CF	Partial cecectomy	Intussusception
Shome et al (1995) [10]	33	F		CF	Ileocecal resection	Endometriosis
So et al (1995) [11]	42	M		CF	Laparoscopic appendectomy	Angiodysplasia
Morales et al (1997) [12]	60	M		CF	Laparoscopic appendectomy	Appendicitis
Gupta et al (2000) [13]	9	M		CF	Partial cecectomy	Intussusception
Kyokane et al (2001) [14]	76	F		Angiography	Transcatheter arterial embolization, conventional appendectomy	Angiodysplasia
Monaghan and Cogbill (2002) [15]	66	M		US, CT	Conventional appendectomy, AAAresection	Primary aortoappendiceal fistula, appendicitis
Lima et al (2004) [16]	16	M	Aspirin 200mg/day	CF	Conventional appendectomy	Crohn’s disease
Rivera-Irigoin et al (2005) [17]	51	M		CF	Conventional appendectomy	Aspirin-induced ulcer
Yamazaki et al (2006) [18]	53	F		CF, CT	Laparoscopic appendectomy	Appendicitis
Ogi et al (2006) [19]	44	M		CF	Laparoscopic appendectomy	Hematoma
Kim et al (2007) [20]	56	M	Few tablets of NSAID	CF	Right hemicolectomy	GIST
Kuntanapreeda (2008) [21]	20	M		CF	Conventional appendectomy, partial cecectomy	Tuberculosis
Baek (2010) [22]	42	M		CT, CF	Laparoscopic appendectomy	Mucosal erosion
Our case	71	M	Ticlopidine hydrochloride	CT, CF	Therapeutic barium enema, laparoscopic appendectomy	Ulcer

Up to now, an emergency operation, including partial cecectomy and hemicolectomy, is generally conducted to control the bleeding in most cases, but no non-operative therapeutic strategy for bleeding from the appendix has yet been established. Only one case of the embolization of the responsible artery has so far been reported [14]. As the therapeutic use of a barium enema has recently been shown to be a useful procedure to treat diverticular bleeding [23,24], we thought that the use of a therapeutic barium enema could also be a practical and less invasive option for controlling such intractable appendix bleeding. In fact, the present case is the first reported case in which a therapeutic barium enema successfully controlled such bleeding.

The mechanism underlying this effect was speculated to be protection of the intestinal epithelia, compression of the blood vessels, coagulating action and the production of a thrombus by the barium itself. In the current case, such functions of the barium enema appeared to be effective for controlling the bleeding from the appendix. From this perspective, the therapeutic barium enema is thought to be useful for the treatment of appendiceal bleeding caused by erosions or ulcers in the appendix, as well as that caused by other disorders, such as diverticulitis and angiodysplasia. We were apprehensive that the therapeutic barium enema might cause the obstruction of the appendix, leading to severe appendicitis. However, the accumulation of barium was almost completely eliminated by the next day, and an appendectomy was successfully performed. An appendectomy is a routine laparoscopic procedure that poses much less risk and less invasive for the patient than an emergency operation, such as cecectomy and hemicolectomy, and arterial embolization. Therefore, the use of a therapeutic barium enema is thought to be a practical and safe procedure to control bleeding from the appendix and to avoid an emergency operation.

### Consent

The patient has given their consent for the case report to be published. Written informed consent was obtained from the patient for publication of this case report and any accompanying images. A copy of the written consent is available for review by the Editor-in-Chief of this journal.

## Competing interest

Tha authors declare that they have no competing interests.

## Authors’ contributions

The work presented here was carried out in collaboration between all authors. All authors have contributed to, seen and approved the manuscript.

## Pre-publication history

The pre-publication history for this paper can be accessed here:

http://www.biomedcentral.com/1471-230X/13/152/prepub
